# QTL Mapping and Candidate Gene Prediction for Crude Protein Content in Sweetpotato (*Ipomoea batatas* (L.) Lam.)

**DOI:** 10.3390/plants15101522

**Published:** 2026-05-16

**Authors:** Donglan Zhao, Jie Wang, Lingxiao Zhao, Shizhuo Xiao, Xibin Dai, An Zhang, Rui Yuan, Yao Wang, Qinglian Li, Tong Ning, Zhilin Zhou, Qinghe Cao

**Affiliations:** Xuzhou Institute of Agricultural Sciences in Jiangsu Xuhuai District, Key Laboratory of Biology and Genetic Breeding of Sweetpotato, Ministry of Agriculture and Rural Affairs, Xuzhou 221131, China; 19991006@jaas.ac.cn (D.Z.);

**Keywords:** sweetpotato, crude protein content, QTL mapping, candidate gene, GWAS

## Abstract

Sweetpotato (*Ipomoea batatas* (L.) Lam.) is an important multifunctional crop with great value in food supply, industrial processing and bioenergy utilization. Crude protein content (CPC) is a core target trait for sweetpotato quality breeding. To dissect the genetic basis of CPC and identify key candidate genes, we used an F_1_ population of 212 individuals. CPC was measured by near-infrared reflectance spectroscopy (NIRS) in 2020 and 2021, and QTL mapping was performed using a high-density SNP genetic linkage map. Candidate genes were explored via a genome-wide association study (GWAS), multiple-database functional annotation, and quantitative real-time PCR (qPCR) validation. The results showed that: (1) CPC in the population exhibited a continuous normal distribution with high inter-year stability, and phenotypic variation was mainly controlled by genetic factors; (2) one stable minor-effect QTL for CPC, *qCPC09-1*, was mapped to Chr09: 7906895–8614924 bp, explaining 5.7% of phenotypic variation; (3) GWAS detected no significant SNP loci, suggesting that CPC is regulated by multiple minor-effect genes; (4) genes within the *qCPC09-1* interval were significantly enriched in three protein synthesis-related KEGG pathways: ribosome, nitrogen metabolism and ubiquinone and other terpenoid–quinone biosynthesis; (5) qPCR verified that *itf09g13420* and *itf09g13230* were upregulated in the low-CPC parent Yushu 10 and negatively correlated with CPC, while *itf09g13550* was upregulated in the high-CPC parent Xin 24 and positively correlated with CPC. These three genes exhibited expression patterns highly consistent with phenotypic differences. This study provides a theoretical basis and technical support for molecular marker-assisted breeding and elite germplasm innovation in sweetpotato.

## 1. Introduction

Sweetpotato (*Ipomoea batatas* (L.) Lam.) is a globally important food and economic crop with strong adaptability, high yield, and rich nutrition. It plays an irreplaceable strategic role in ensuring food security, promoting industrial development, and improving residents’ dietary structure. With the popularization of health-conscious consumption, genetic improvement in nutritional quality has become a major focus of sweetpotato breeding. Sweetpotato storage roots are rich in complex carbohydrates, high-quality plant-derived proteins, mineral elements such as iron (Fe) and calcium (Ca), and dietary fiber that promotes digestion [1]. Its protein contains a complete set of 18 amino acids, including all eight essential amino acids for humans, with contents higher than those of many plant proteins, showing unique nutritional value and bioactive potential [2]. Studies have shown that sweetpotato protein has potential health-promoting effects in cancer prevention [3], diabetes management [4], and blood pressure regulation [5]. Therefore, dissecting the genetic basis of CPC, mapping key QTLs, and mining candidate genes are of great theoretical and practical significance for precise quality improvement and accelerated elite breeding of sweetpotato.

As a hexaploid crop (2n = 6x = 90), sweetpotato has a complex genome characterized by high heterozygosity and intraspecific cross-incompatibility, which severely restricts the fine mapping and functional analysis of quality-related genes. Limited by its complex genetic background, QTL mapping of sweetpotato lags behind that of other crops. Previous studies mostly focused on starch content [6,7,8,9], dry matter content [8,10], and β-carotene content [8,11], using traditional PCR-based markers such as AFLP, SRAP, and SSR [6,7,8,9,10,11]. These markers have limitations such as low polymorphism, limited genome coverage, and poor stability, failing to meet the requirements of high-density genetic map construction. In contrast, single nucleotide polymorphism (SNP), the third-generation molecular marker, has become ideal for high-density genetic mapping due to its abundance, wide distribution, high stability, and automated genotyping. The first SNP linkage map of sweetpotato was constructed by Shirasawa et al. [12], containing 96 linkage groups with an average marker distance of 1.18 cM. Later, Yan et al. [13] used a high-density SNP map to map 10 QTLs related to skin color, flesh color, and anthocyanin content. Our team previously constructed an F_1_ population of 212 individuals and developed high-throughput SNPs via SLAF-seq (Specific-Locus Amplified Fragment Sequencing), successfully building a high-density genetic linkage map covering 2441.56 cM with an average marker interval of 0.51 cM [14], laying a foundation for genetic dissection of important traits in sweetpotato.

Phenotyping is a critical step for QTL mapping. Traditional chemical methods are time-consuming, costly, destructive, and involve chemical reagents, making them unsuitable for large-scale, multi-environment precise phenotyping. As a rapid, accurate, non-destructive, and eco-friendly technique, near-infrared reflectance spectroscopy (NIRS) has been widely used for quantitative analysis of various nutrients in agricultural products [15]. It requires minimal sample preparation and can simultaneously determine multiple components in one scan, making it suitable for high-throughput phenotyping of large populations. Studies have confirmed that NIRS can accurately measure CPC [16,17,18,19], starch, soluble sugar, total nitrogen, β-carotene, iron, zinc, magnesium, and other nutrients in sweetpotato [15,18,19,20,21].

To date, QTL mapping and genetic dissection for sweetpotato CPC have not been reported, and its genetic basis, genetic effect, and regulatory genes remain largely unclear. Therefore, this study used the previously constructed F_1_ population, conducted precise CPC phenotyping for two consecutive years by NIRS, and performed QTL mapping combined with the high-density SNP map. The results will lay a foundation for candidate gene mining and functional analysis, and provide theoretical support for marker-assisted selection and elite germplasm creation in sweetpotato.

## 2. Results

### 2.1. Analysis of CPC in the Mapping Population

A two-way ANOVA was performed to evaluate the effects of parent line (Xin 24 and Yushu 10) and year (2020 and 2021) on CPC value. The results (Table 1) showed significant main effects for parent line (F_1,8_ = 382.48, *p* < 0.001) and year (F_1,8_ = 19.74, *p* = 0.002). Importantly, the interaction between parent line and year was also significant (F_1,8_ = 132.84, *p* < 0.001), indicating that the CPC values of the two parent lines changed differently across the two years.

**Figure 1:**
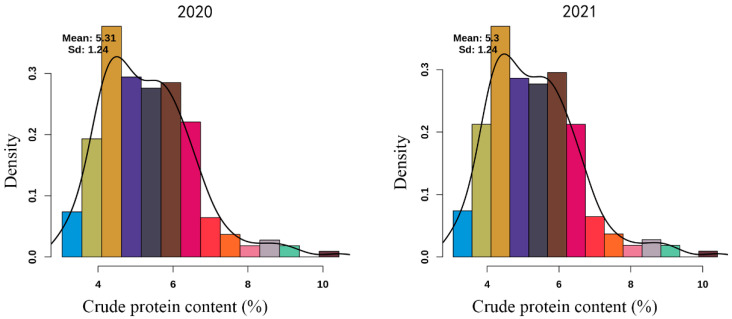
Distribution of crude protein content in the F_1_ population in 2020 and 2021.

CPC of the F_1_ population showed a continuous near-normal distribution in both 2020 and 2021, with highly consistent distribution patterns across years (Figure 1). In 2020, the mean CPC was 5.31% with a standard deviation (SD) of 1.24%; in 2021, the mean was 5.30% with SD = 1.24%. The phenotypic peak was concentrated in 4–6%, with few individuals below 3% or above 8%. The stable normal distribution indicated that environmental effects were small and genetic factors dominated phenotypic variation, providing a solid basis for QTL mapping.

### 2.2. QTL Mapping for CPC

**Figure 2:**
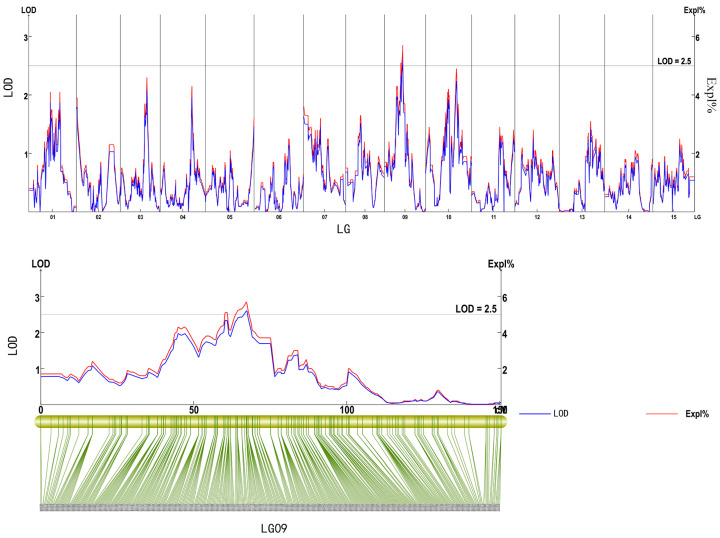
QTL analysis of crude protein content on the linkage map. The green lines represent the SNP markers distributed along linkage group 09.

One consistent QTL *qCPC09-1* for CPC was mapped to linkage group 9 (LG09) based on phenotypic values in 2020, 2021, and their average. It was located in the physical interval Chr09: 7906895–8614924 bp, with an LOD score of 2.61 and a phenotypic contribution rate of 5.7% (Table 2, Figure 2). Although the phenotypic variance explained by qCPC09-1 is relatively low, its consistent detection across two individual years and the two-year average dataset indicates genuine inter-annual stability. In polyploid crops such as sweetpotato, where genetic redundancy and dosage effects are common, minor-effect QTLs often represent authentic genetic determinants rather than spurious noise. The detection of a stable albeit minor QTL is consistent with the quantitative nature of CPC and provides a reproducible entry point for further fine mapping.

### 2.3. GWAS for CPC

**Figure 3:**
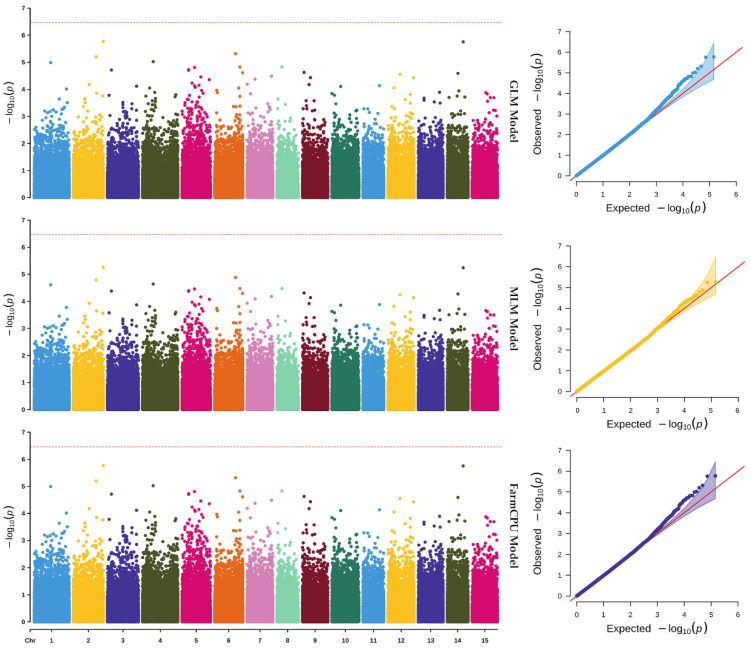
Manhattan plots (**left panels**) and Q-Q plots (**right panels**) of GWAS results for crude protein content in sweetpotato, based on three statistical models: GLM, MLM and FarmCPU. The red dashed line is significance threshold of 5% Bonferroni correction.

A GWAS using GLM, MLM, and FarmCPU models detected no significant SNP loci exceeding the genome-wide threshold for CPC. The association signals were evenly distributed across chromosomes, and the Q-Q plots showed no early deviation of observed values from expected value, indicating that the lack of significant associations is not due to population stratification or model misspecification. These results suggest that CPC is regulated by multiple minor-effect genes rather than a few major genes (Figure 3).

### 2.4. Functional Annotation and Candidate Gene Prediction in the QTL Interval

Functional annotation of 104 genes in *qCPC09-1* was performed using NR, COG, GO, and KEGG databases. NR annotation showed most homologs belong to Solanaceae species, mainly *Nicotiana sylvestris* (30.8%) and *Nicotiana tomentosiformis* (25.0%). COG classification revealed enrichment in amino acid transport and metabolism (E), carbohydrate transport and metabolism (G), and translation, ribosomal structure and biogenesis (J). GO terms were enriched in catalytic activity, binding, metabolic processes, and cellular processes.

**Figure 4:**
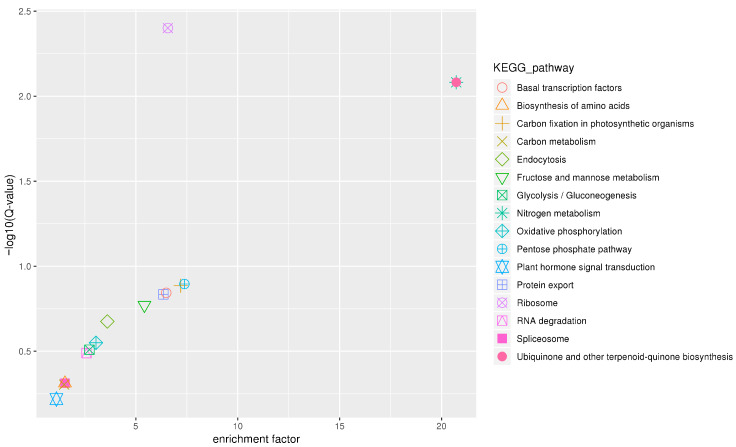
KEGG pathway enrichment analysis of annotated genes within the *qCPC09-1* interval. The Benjamini–Hochberg method was applied to control the false discovery rate (FDR), and Q-values (adjusted *p*-values) were calculated. Pathways with Q-value < 0.05 were considered significantly enriched.

KEGG pathway analysis identified three significantly enriched pathways (Q-value < 0.05): ribosome, nitrogen metabolism, and ubiquinone and other terpenoid–quinone biosynthesis. Additional enrichment was observed in protein export and biosynthesis of amino acids, and other related pathways (Figure 4). These results indicate that genes in the *qCPC09-1* interval are primarily involved in amino acid metabolism and protein synthesis, consistent with their potential role in regulating crude protein content. Based on these annotations, eight candidate genes were primarily selected (Table 3), four of which were further validated by qRT-PCR.

### 2.5. Expression Analysis of Candidate Genes

**Figure 5:**
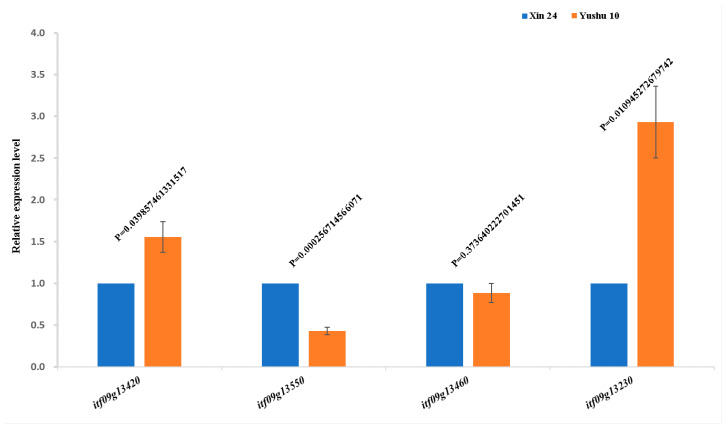
Relative expression of crude protein content candidate genes in the two parents. Error bars indicate standard error (SE). *p*-value from Student’s *t*-test.

qRT-PCR validation revealed that *itf09g13230* and *itf09g13420* were significantly upregulated in low-CPC parent Yushu 10, with expression levels 2.93-fold and 1.55-fold higher than those in high-CPC parent Xin 24, indicating a negative correlation with crude protein content. By contrast, *itf09g13550* was markedly downregulated in Yushu 10, with its expression level only 0.43-fold of that in Xin 24, showing a positive correlation with crude protein accumulation. No significant expression difference in itf09g13460 was detected between the two parents (Figure 5).

## 3. Discussion

The complex genetic background of sweetpotato, as a highly heterozygous hexaploid crop, together with self-incompatibility, has long hindered the genetic dissection of important quality traits such as crude protein content [22]. To our knowledge, this study represents the first report on QTL mapping for crude protein content (CPC) in sweetpotato using a high-density SNP genetic linkage map. Our phenotypic results demonstrated that CPC in the F1 population showed a continuous, near-normal distribution across two years, with highly stable mean values and standard deviations, indicating that CPC is a typical quantitative trait controlled by multiple genes rather than a single major gene. This pattern is consistent with the genetic architecture of starch content in sweetpotato [6]. The strong inter-year stability also suggests that phenotypic variation was mainly driven by genetic factors, with relatively minor environmental interference, providing a solid foundation for reliable QTL detection.

In the present study, only one minor-effect QTL, *qCPC09-1*, was detected on chromosome 9, explaining 5.7% of the phenotypic variation. Notably, no significant SNP loci were identified by GWAS using three commonly adopted models (GLM, MLM, FarmCPU). This discrepancy may be attributed to the fundamental differences in statistical power and resolution between linkage and association mapping. QTL analysis, based on a biparental population with limited recombination events, is more sensitive to loci with minor effects, especially when the genetic background is controlled. In contrast, GWAS relies on historical recombination in natural populations and requires very strict significance thresholds to control false positives (e.g., Bonferroni correction), which can easily mask minor-to-moderate-effect loci in polyploid genomes with high linkage disequilibrium (LD). These results collectively support the hypothesis that CPC in sweetpotato is regulated by numerous minor-effect polygenes, rather than by one or several major genes. This genetic architecture is typical in hexaploid species, where duplicated genomic regions and dosage effects dilute the effect of individual loci. Similar results have been reported in other polyploid crops, where complex nutritional traits are often controlled by a large number of small-effect loci distributed across the genome [23]. Therefore, QTL mapping remains more effective than GWAS for capturing moderate-effect loci in such genetic backgrounds.

The physical interval of *qCPC09-1* spans approximately 700 kb and contains 104 annotated genes. Functional annotation of the 104 genes within the *qCPC09-1* interval using multiple databases revealed that genes in this region are enriched in pathways directly related to protein synthesis, such as amino acid transport and metabolism, translation, and ribosome structure and biogenesis. Further KEGG pathway mapping identified three significantly enriched pathways: ribosome, nitrogen metabolism, and ubiquinone and other terpenoid–quinone biosynthesis. Additional enrichment was observed in protein export and biosynthesis of amino acids, and other related pathways, strongly indicating that this QTL interval is a crucial genetic region regulating crude protein synthesis and accumulation in sweetpotato. Meanwhile, the high homology of these genes to *Nicotiana* and other *Solanaceae* species indicates evolutionary conservation of protein metabolism pathways, which facilitates cross-species reference for subsequent functional studies.

From the 104 genes within *qCPC09-1*, we prioritized eight candidate genes based on functional annotation, and further validated four of them using qRT-PCR. Among these, three genes showed expression patterns highly consistent with CPC phenotypic differences between the two parents, indicating their strong potential as regulators of protein content.

The gene *itf09g13550* encodes the 60S ribosomal protein L10a-1 (RPL10a-1), a component of the eukaryotic ribosome large subunit and also a key factor in the formation of functional 80S ribosomes [24,25]. Ribosomal proteins are indispensable for ribosome assembly, mRNA translation, and protein biosynthesis. Studies have shown that RPL10 family genes exhibit no functional redundancy and are involved in translation during plant development and UV-B stress [25]. RPL10A exhibits the highest expression level during germination and early developmental stages and acts as a positive regulator of abscisic acid (ABA)-dependent responses in Arabidopsis [26]. Whether these ribosomal protein genes are also associated with crude protein content in sweetpotato storage roots remains to be investigated. In this study, *itf09g13550* was significantly upregulated in the high-protein parent Xin 24, and downregulated in the low-protein parent Yushu 10, showing a positive correlation with crude protein content. This expression pattern strongly suggests that *itf09g13550* promotes protein synthesis by enhancing ribosome function or translational efficiency, thereby increasing protein accumulation in sweetpotato storage roots. The regulatory mechanism of this gene on crude protein accumulation in sweetpotato storage roots remains to be further elucidated via gene cloning, transgenic complementation, and functional characterization.

In contrast, *itf09g13420* and *itf09g13230* exhibited negative correlation with CPC. The gene *itf09g13420* encodes a chloroplast-localized 50S ribosomal protein L20, which participates in the plastid protein translation system. Chloroplasts are semi-autonomous organelles that synthesize many proteins related to photosynthesis and energy metabolism [27]. Plastid ribosomal proteins are essential for plants, function in diverse aspects of plant biology, and are closely related to plant tolerance to adverse environmental conditions [28,29]. Although chloroplast proteins contribute only a small portion of total crude protein in storage roots, their dysfunction may disrupt carbon–nitrogen balance, indirectly reducing nitrogen allocation to storage protein synthesis. The high expression of *itf09g13420* in Yushu 10 may therefore reflect a compensatory response to impaired plastid function or redirected metabolic flux, leading to lower storage protein accumulation.

Notably, *itf09g13230* encodes a Pro-X carboxypeptidase-like protein, which is homologous to vacuolar peptidases involved in protein degradation and peptide processing. Lysosomes are key organelles responsible for substance degradation, metabolic regulation, and signal transduction in eukaryotic cells. Unlike animal cells, plant cells lack typical lysosomes, and their lysosome-like degradative functions are mainly performed by vacuoles [30,31]. Existing studies have shown that plant carboxypeptidase-like proteins are mainly localized in vacuoles and participate in intracellular protein degradation, peptide processing, stress response, and the regulation of growth and development [32,33]. The extremely high expression of this gene in the low-protein parent Yushu 10 (nearly three-fold that of Xin 24) strongly suggests that enhanced protein degradation contributes to reduced crude protein content. To date, few studies have linked carboxypeptidase-like genes to storage protein regulation in root crops; our findings therefore provide a novel perspective for understanding protein content determination in sweetpotato.

Although this study successfully identified a stable QTL and three promising candidate genes, some limitations should be noted. First, in this study, three phenotypic datasets (2020, 2021, and their two-year average) were independently used for QTL mapping. The same QTL, *qCPC09-1*, was consistently detected across all three datasets, indicating good inter-annual stability. Nevertheless, we acknowledge that two years of data are insufficient to fully assess its stability across broader environmental conditions. Future work should include multi-environment QTL mapping across additional locations and years to further validate its stability. Second, the QTL interval still contains 104 genes, requiring fine mapping to narrow down the QTL interval. Third, the functions of *itf09g13550*, *itf09g13420*, and *itf09g13230* remain to be verified through transgenic complementation, gene editing, or gene silencing. Future studies may expand the population size, integrate multi-environment trials, and combine bulked segregant analysis (BSA) with whole-genome resequencing to identify more minor-effect QTLs and construct a comprehensive regulatory network for CPC. In summary, this study provides the first genetic mapping evidence for crude protein content in sweetpotato, revealing its polygenic, minor-effect inheritance pattern. We identified a stable minor-effect QTL *qCPC09-1* and three key candidate genes that may regulate protein synthesis and degradation. These results provide a preliminary foundation for marker-assisted selection, gene functional research, and genetic improvement in protein nutritional quality in sweetpotato breeding, offering initial insights rather than definitive solutions.

## 4. Materials and Methods

### 4.1. Plant Materials and Genetic Linkage Map

The mapping population consisted of 212 F_1_ individuals derived from a cross between Xin 24 (high CPC) and Yushu 10 (low CPC). The population was planted in the experimental field of Xuzhou Sweetpotato Research Center in 2020 and 2021, with 10 plants per entry. Row spacing was 80 cm, plant spacing 25 cm, and field management followed local conventional practices. Storage roots were harvested about 120 days after transplanting.

Genetic linkage mapping was performed using two parents and 212 progeny individuals. The two parents were subjected to whole-genome resequencing, and the 212 progenies were genotyped using SLAF-seq. Genotyping was conducted based on the reference genome of *Ipomoea trifida*, a wild relative of sweetpotato. A total of 5664 SNP markers were initially screened, and 4758 high-quality SNPs were finally anchored onto the genetic linkage map. The total distance of the genetic map was 2441.56 cM, with an average marker interval of 0.51 cM across 15 linkage groups (LGs).

The number of mapped SNPs per linkage group ranged from 185 (LG08; total length = 141.42 cM; average interval = 0.77 cM) to 454 (LG01; total length = 174.61 cM; average interval = 0.39 cM). Among all LGs, LG08 was the shortest and contained the fewest markers, whereas LG06 was the longest, with 421 markers, a total length of 180.88 cM, and an average marker interval of 0.43 cM. The largest gap between adjacent markers was observed in LG04 (19.21 cM), and the smallest gap was in LG09 (5.58 cM). In terms of marker density, LG14 was the sparsest (225 markers; average interval = 0.79 cM), while LG09 was the densest (390 markers; average interval = 0.39 cM).

Population curation and marker analysis procedures were carried out according to the method described in our previously published research [14].

### 4.2. Measurement of Crude Protein Content

CPC was determined by NIRS [17]. Five medium-sized, pest-free storage roots were selected per sample, washed, dried, peeled, sliced, and vacuum-freeze-dried at −50 °C for 72 h. The samples were ground into powder and sieved (100 mesh). Scanning was performed using a VECTOR22/N Fourier transform near-infrared reflectance spectrometer (BRUKER, Karlsruhe, Germany) with three technical replicates per sample.

### 4.3. QTL Mapping

QTL analysis for CPC was conducted using the high-density SNP map [14] and the R/qtl package (v1.52). The significance threshold of LOD scores was determined by a permutation test (*p* < 0.05) with 1000 permutations at the 5% significance level. Composite interval mapping (CIM) was used to detect significant QTLs. Scanning was performed every 1 cM on each linkage group to calculate the phenotypic contribution rate.

### 4.4. Genome-Wide Association Study (GWAS)

A GWAS was performed using the rMVP package (v1.0.6) in R with three models: general linear model (GLM), mixed linear model (MLM), and fixed and random model circulating probability unification (FarmCPU). Both MLM and FarmCPU included principal component analysis (PCA) and kinship matrix (Kinship) as covariates to control population structure and genetic background. The significance threshold was set as −log_10_(0.05/total number of SNPs). Manhattan plots and Q-Q plots were generated for visualization.

### 4.5. Function Annotation and KEGG Pathway Enrichment Analysis

The candidate genes were functionally annotated by alignment against the COG, GO, KEGG and NR databases using standard bioinformatics pipelines. For COG and GO classification, genes were assigned to functional categories based on sequence homology.

KEGG pathway enrichment analysis was performed using the hypergeometric test to identify significantly enriched pathways. All annotated genes in the *Ipomoea trifida* reference genome were used as the background gene set. The Benjamini–Hochberg method was applied to control the false discovery rate (FDR), and Q-values (adjusted *p*-values) were calculated. Pathways with Q-value < 0.05 were considered significantly enriched. The results were visualized using a bubble plot, where the *x*-axis represents the enrichment factor and the *y*-axis represents the −log_10_(Q value).

### 4.6. Candidate Gene Screening and qRT-PCR Analysis

Based on the QTL interval and reference genome annotation, all genes in the target region were extracted. Homologous alignment was performed using the NCBI database. Candidate genes related to CPC were screened by integrating functional annotation, pathway analysis, and expression patterns.

Total RNA was extracted from fully expanded functional leaves of greenhouse-grown potted seedlings 90 days after planting. For two parents, three independent biological replicates were used, and each biological replicate consisted of a pool of three individual plants to reduce individual variation. Three technical replicates were performed for each biological replicate. Subsequently, cDNA was synthesized using a reverse transcription kit. Primers were designed based on the CDS sequences of candidate genes for qRT-PCR. The reaction system (20 μL) contained 10 μL SYBR Green mix, 20 ng cDNA, 1 μL of each forward and reverse primer, and ddH2O. The amplification program was: 95 °C for 1 min; 40 cycles of 95 °C for 15 s, 60 °C for 15 s, 72 °C for 20 s. Expression levels were normalized against the internal control gene *Actin* [34] of sweetpotato using the 2^(−ΔΔCt)^ method. The expression stability of Actin was evaluated using geNorm and ΔCt algorithms to ensure reliable normalization. An independent sample *t*-test was used to compare gene expression between the two parents, with significance levels indicated as * *p* < 0.05 and ** *p* < 0.01.

## 5. Conclusions

This study provides the first systematic genetic dissection of crude protein content (CPC) in sweetpotato. The results demonstrate that CPC is governed by multiple minor-effect polygenes rather than major loci, with *qCPC09-1* representing a stable but minor QTL explaining only 5.7% of the phenotypic variation. Notably, three candidate genes—*itf09g13550* (putative positive regulator) and *itf09g13420*/*itf09g13230* (putative negative regulators)—were identified, revealing a complex regulatory network involving protein metabolism. Although the QTL effect is minor, its inter-annual stability and the consistency among phenotypic distribution and QTL mapping collectively support the conclusion that CPC in sweetpotato is controlled by a polygenic architecture with minor-effect loci. These findings provide a foundation for future fine mapping, candidate gene validation, and marker-assisted breeding.

## Figures and Tables

**Table 1:** Two-way ANOVA results for the effects of parent line and year on CPC value.

Source of Variation	SS	df	MS	F	*p*-Value
Year	0.05253	1	0.05253	19.73935	0.00216
Parent	1.017831	1	1.017831	382.4756	4.86 × 10^−8^
Year × Parent	0.353513	1	0.353513	132.8414	2.91 × 10^−6^
Within (Error)	0.021289	8	0.002661		
Total	1.445164	11			

**Table 2:** QTLs for crude protein content in sweetpotato.

QTL	Linkage Group	Marker Interval	Marker Number	Physical Interval (bp)	LOD	Expl (%)	No. of Genes
*qCPC09-1*	LG09	Marker2165975—Marker2173321	9	Chr09 7906895 8614924	2.61	5.7	104

**Table 3:** Annotation of key candidate genes in the QTL interval.

Candidate Genes	Physical Location	COG Annotation	NR Function Annotation
*itf09g13420*	Chr 09: 8183546-8186845	[J] Translation, ribosomal structure and biogenesis	PREDICTED: 50S ribosomal protein L20, chloroplastic [Sesamum indicum]
*itf09g13550*	Chr 09: 8278898-8280782	[J] Translation, ribosomal structure and biogenesis	PREDICTED: 60S ribosomal protein L10a-1 [Glycine max]
*itf09g13380*	Chr 09: 8141942-8144040	[J] Translation, ribosomal structure and biogenesis	hypothetical protein CICLE_v10013022mg [Citrus clementina]
*itf09g13460*	Chr 09: 8198982-8211045	[U] Intracellular trafficking, secretion, and vesicular transport	PREDICTED: signal recognition particle 54 kDa protein, chloroplastic [Solanum lycopersicum]
*itf09g13230*	Chr 09: 7970488-7980561	--	PREDICTED: lysosomal Pro-X carboxypeptidase-like [Nicotiana sylvestris]
*itf09g13270*	Chr 09: 8014594-8015554	[K] Transcription	PREDICTED: transcription initiation factor TFIID subunit 9 isoform X1 [Solanum tuberosum]
*itf09g13580*	Chr 09: 8289714-8291767	--	PREDICTED: protein ABSCISIC ACID-INSENSITIVE 5 isoform X1 [Solanum lycopersicum]
*itf09g13930*	Chr 09: 8609072-8611533	[G] Carbohydrate transport and metabolism	PREDICTED: fructose-bisphosphate aldolase cytoplasmic isozyme [Nicotiana tomentosiformis]

Note: “--” = No annotation information available in the corresponding database.

## Data Availability

The original contributions presented in the study are included in the article, further inquiries can be directed to the corresponding authors.
